# Early neurosyphilis presenting with facial palsy and an oral ulcer in a patient who is human immunodeficiency virus positive: a case report

**DOI:** 10.1186/s13256-017-1297-0

**Published:** 2017-05-13

**Authors:** Evangeline Njiru, Jamil Abdulkadir, Zipporah Kamuren, Gabriel Kigen

**Affiliations:** 1 Department of Internal Medicine, Moi University School of Medicine, P.O. Box 4606, 30100 Eldoret, Kenya; 2 Department of Pharmacology and Toxicology, Moi University School of Medicine, P.O. Box 4606, 30100 Eldoret, Kenya

**Keywords:** Case report, Neurosyphilis, HIV, CNS, Diagnosis

## Abstract

**Background:**

Neurosyphilis is the tertiary stage of *Treponema pallidum* infection that involves the central nervous system, which occurs within days or weeks after an initial syphilis infection, especially in immunocompromised patients. The diagnosis of neurosyphilis is quite challenging as it is uncommon and often presents with obscure symptoms since any organ system may be involved.

**Case presentation:**

We describe a case of a 40-year-old African man who is human immunodeficiency virus positive with early neurosyphilis who presented with a stiff neck, headache, confusion, restlessness, and a left-sided chest pain; he did not respond to an empiric treatment of ceftriaxone and fluconazole for meningitis, and tramadol for headache. Ten days after admission, he developed generalized tonic–clonic convulsions; on examination he had ipsilateral facial nerve palsy and an oral ulcer, and responded well to benzathine penicillin treatment.

**Conclusions:**

Laboratory diagnosis of neurosyphilis is challenging because to date there is no single laboratory test which is considered sensitive enough for diagnosis of the disease, especially in resource-limited settings. Clinical judgment is still an important part of diagnosis; and neurosyphilis should be considered a diagnostic differential in patients with Human Immunodeficiency Virus presenting with central nervous system involvement and in other high-risk patients.

## Background

Neurosyphilis is the tertiary stage of *Treponema pallidum* infection that involves the central nervous system (CNS). It is marked by the presence of treponemes in the CNS with associated cellular and biochemical changes including raised cerebrospinal fluid (CSF) proteins and cell count. It can occur within days or weeks after an initial syphilis infection, especially in immunocompromised patients, including those with human immunodeficiency virus (HIV) which has been postulated to alter the course of syphilis infection [1, 2]. Despite CNS involvement, many patients do not develop neurological manifestations at early stages, but symptomatic neurosyphilis has been noted to occur more commonly in patients with HIV [3]. CNS invasion is largely limited to the meninges with symptoms such as headache, neck stiffness, and cranial nerve paresis. Parenchymal CNS involvement is accompanied by grave effects such as stroke, tabes dorsalis, and general paresis [3, 4].

If there is early detection of primary syphilis then it responds well to treatment, but diagnosis is often complicated as culturing of treponemes is difficult, and current methods largely rely on immunologic responses and microscopic visualization of the organisms from the exudates collected from an active chancre [5–7]. The diagnosis of neurosyphilis is even more challenging as it is not common despite the resurgence of HIV-associated syphilis, and often presents with obscure symptoms since any organ system may be involved [1, 8]. It has been described to present with nonspecific clinical symptoms such as depression and sudden blindness [9]; mild dementia, chronic chorioretinitis, hearing loss, and bilateral hippocampal atrophy [10, 11]; optic neuritis [12–14], hemorrhagic exudative optic neuropathy [15], hemiparesis, and Jacksonian epilepsy [16]; acute stroke [17], or stroke-like syndrome presenting as mixed dysphasia, right upper motor neuron facial palsy, and right hemiparesis [18]. The wide range of clinical manifestations of neurosyphilis therefore quite often leads to delayed or even in some instances missed diagnosis and treatment.

We describe a case of a 40-year-old African man who is HIV positive with early neurosyphilis presenting with headache, facial nerve palsy, and an oral ulcer who responded to benzathine penicillin treatment.

## Case presentation

A 40-year-old African man who is HIV positive presented to our hospital with a 1-day history of confusion, headache, restlessness, and a left-sided chest pain. He had been diagnosed 2 months earlier as having acute meningitis during which time it was discovered that he was also HIV positive from a routine test carried out at the time of admission. He was then empirically treated for both bacterial and cryptococcal meningitis with ceftriaxone 2 gm once a day for 10 days and high-dose fluconazole (800 mg) for 2 weeks. In addition, he was enrolled into HIV care, and started on a highly active antiretroviral therapy (HAART) regimen consisting of a daily dose of a tablet containing a combination of tenofovir (300 mg), lamivudine (300 mg), and efavirenz (600 mg), as well as co-trimoxazole 960 mg once a day for prophylaxis. His CD4 count done at the initiation of treatment was 359 cells/mm^3^ (15.4%). He was later discharged and given antibiotics to be administered orally (cefuroxime 500 mg twice a day for 5 days and continued on co-trimoxazole 960 mg once a day) as well as tramadol 100 mg once a day for the treatment of a throbbing headache that he was still complaining of.

Three months later, he was readmitted with complaints of an acute onset headache and confusion for 1 day. He also complained of a left-sided chest pain, but had no history of coughing. On examination, he scored a Glasgow Coma Scale (GCS) of 15/15 and was oriented in time, place, and person. However, he had a stiff neck with a positive Kernig’s sign but with no focal neurological deficit appreciated. His vital signs were within normal range, with blood pressure of 110/90 mmHg, pulse of 83 beats/minute, temperature of 36.8 °C, and blood oxygen saturation (SPO_2_) of 96%. On admission, all parameters of his full hemogram were within normal range, except for a mild leukopenia of 3.1×10^9^/L (normal range 4.3 to 11×10^9^/L) and a lymphocyte count of 0.8×10^9^/L (range 1.5 to 4×10^9^/L), but with a normal absolute neutrophil count of 1.6×10^9^/L (1 to 4.6×10^9^/L). His hemoglobin level was within normal range at 12.8 g/dl, with red cell mean corpuscular volume (MCV) and mean corpuscular hemoglobin (MCH) indices of 75.8 fL and 24.1 pg respectively. His platelets were adequate at 247×10^9^/L. Serum urea and creatinine levels were normal at 2.97 mmol/L and 93 μmol/L respectively. His sodium, potassium, and chloride levels were also normal at 133.1 mmol/L, 4.33 mmol/L, and 97.9 mmol/L respectively. A lumber puncture revealed CSF under high pressure, but it was clear with no coagulation. CSF results indicated raised proteins of 204.7 mg/dL (normal 15 to 45 mg/dL), white blood cell (WBC) count of 3 cells/μl, red blood cell (RBC) count of 0 cells/μl, and normal glucose levels. India ink was negative for *Cryptococcus* and a cryptococcal antigen (CrAg) test was equally negative. A Gram stain did not reveal any bacteria, and there was no growth of bacteria or yeast on CSF culture. A GeneXpert assay on CSF did not reveal *Mycobacterium tuberculosis*. His chest X-ray was normal. A computed tomography (CT) scan of his head showed an ill-defined non-enhancing hypodense subcortical lesion at the right frontal lobe suspicious for cerebritis, with subtle meningeal enhancement. A standard 12-lead electrocardiogram (ECG) showed features suggestive of left ventricular hypertrophy (LVH) based on a positive voltage criterion for LVH with left axis shift. As a result, an echocardiogram was done which showed only mild left ventricular systolic dysfunction with an ejection fraction (EF) of 50% and left ventricular diastolic dysfunction grade 1.

However, he was again empirically managed for both cryptococcal and bacterial meningitis with high-dose fluconazole (800 mg once daily) and ceftriaxone (2 g twice a day), and tramadol 50 mg twice a day for management of his headache. Despite 10 days of treatment, his condition continued to deteriorate, characterized by a fall in the GCS level to 13/15 from the previous 15/15, confusion, violent behavior, disinhibition, mood changes, delusions, and self-neglect. He also developed ipsilateral facial nerve palsy, and generalized tonic–clonic convulsions which were managed with carbamazepine 200 mg twice a day. He sustained a left-sided painful jaw swelling secondary to a convulsive trauma, for which a dental review was sought, and possible left temporomandibular joint dislocation with a suspected subcondylar fracture. Unfortunately, an orthopantomogram (OPG) was not done for him due to financial constraints. However, during the examination of his mouth for the jaw dislocation, an oral ulcer on the right molar region was discovered; but, due to his discomfort and inability to fully cooperate, the examination was limited which precluded detailed and repeated examinations of the ulcer. On further probing, it became apparent that he had had the oral ulcer for several weeks (the exact duration was, however, not clear). An infectious disease consultant suggested the possibility of neurosyphilis based on the presence of acute worsening cognitive impairment, despite coverage for bacterial and cryptococcal meningitis, with the presence of the subacute oral ulcer. Suspicion of tertiary syphilis was further strengthened by the overall clinical picture: tying up together the history of HIV infection; the sudden onset convulsions that were not due to obvious structural brain disease, medications, or systemic illness; and the presence of facial nerve palsy. A Venereal Disease Research Laboratory (VDRL) test on an undiluted sample was negative. Cognizant of the prozone effect observed in serological syphilis testing in the presence of HIV infection, a repeat test at a 1:100 dilution factor was performed. This second test was positive confirming the presence of neurosyphilis. He was subsequently started on a benzathine penicillin regimen of 2.4 MU once a week for 3 weeks. He improved tremendously and was discharged.

## Discussion

There has been a recent worldwide upsurge in incidents of syphilis infections, particularly among patients who are HIV positive. This coupled with the changing clinical presentations has warranted a new focus on the diagnosis and treatment of the disease [17, 19, 20]. The various clinical manifestations of neurosyphilis in HIV/acquired immunodeficiency syndrome (AIDS) in particular, present a diagnostic challenge and delay management of the disease, especially in resource-limited settings [21]. It is complicated by the difficulty in distinguishing between the neurologic disorders attributed to *T. pallidum* and those caused by opportunistic infections [17]. The signs and symptoms of neurosyphilis are protean, and the US Centers for Disease Control and Prevention (CDC) has provided general criteria for classification based on the dominant characteristics of the patient which include neuropathic, meningovascular, and myelopathic symptoms. However, these criteria are too broad to provide an effective guide for diagnosis, and require clinical judgment to reduce diagnostic omissions [22, 23]. In addition, there are several overlaps between the symptoms [24].

Laboratory diagnosis of neurosyphilis is equally challenging. To date, there is no single laboratory test that is considered sensitive enough for diagnosis of the disease and clinical judgment is still the hallmark of diagnosis [25, 26]. The standard diagnostics involve nontreponemal screening followed by CSF treponemal-specific antibody confirmatory tests [27]. The interpretation of the false-negative and false-positive results is, however, challenging as a negative test may not necessarily exclude diagnosis if there is clinical evidence [19, 25].

## Conclusions

The case that we have described is neurosyphilis with an unusual presentation due to the presence of HIV and its management. Medical personnel should heighten their degree of suspicion of syphilis among the HIV population; and neurosyphilis should be considered a diagnostic differential in patients with HIV with CNS involvement and other high-risk patients.

## Abbreviations

AIDS: Acquired immunodeficiency syndrome
CDC: Centers for Disease Control and Prevention
CNS: Central nervous system
CrAg: Cryptococcal antigen
CSF: Cerebrospinal fluid
CT: Computed tomography
ECG: Electrocardiogram
EF: Ejection fraction
GCS: Glasgow Coma Scale
HAART: Highly active antiretroviral therapy
LVH: Left ventricular hypertrophy
MCH: Mean corpuscular hemoglobin
MCV: Mean corpuscular volume
OPG: Orthopantomogram
RBC: Red blood cell
SPO_2_: Blood oxygen saturation
VDRL: Venereal Disease Research Laboratory
WBC: White blood cell
